# Chiral-Selective Tamm Plasmon Polaritons

**DOI:** 10.3390/ma14112788

**Published:** 2021-05-24

**Authors:** Meng-Ying Lin, Wen-Hui Xu, Rashid G. Bikbaev, Jhen-Hong Yang, Chang-Ruei Li, Ivan V. Timofeev, Wei Lee, Kuo-Ping Chen

**Affiliations:** 1 Institute of Imaging and Biomedical Photonics, College of Photonics, National Chiao Tung University, 301 Gaofa 3rd Road, Tainan 71150, Taiwan; e1253400@gmail.com (M.-Y.L.); uxxsh8@gmail.com (W.-H.X.); wlee@nctu.edu.tw (W.L.); 2 Institute of Imaging and Biomedical Photonics, College of Photonics, National Yang Ming Chiao Tung University, 301 Gaofa 3rd Road, Tainan 71150, Taiwan; 3 Kirensky Institute of Physics, Federal Research Center KSC SB RAS, 660036 Krasnoyarsk, Russia; ivan-v-timofeev@ya.ru; 4 Siberian Federal University, 660041 Krasnoyarsk, Russia; 5 Institute of Photonic System, College of Photonics, National Chiao Tung University, 301 Gaofa 3rd Road, Tainan 71150, Taiwan; s87069@hotmail.com; 6 Institute of Photonic System, College of Photonics, National Yang Ming Chiao Tung University, 301 Gaofa 3rd Road, Tainan 71150, Taiwan; 7 Institute of Lighting and Energy Photonics, College of Photonics, National Chiao Tung University, 301 Gaofa 3rd Road, Tainan 71150, Taiwan; g10592000@hotmail.com; 8 Institute of Lighting and Energy Photonics, College of Photonics, National Yang Ming Chiao Tung University, 301 Gaofa 3rd Road, Tainan 71150, Taiwan

**Keywords:** metasurfaces, tamm plasmon polaritons, chirality

## Abstract

Chiral-selective Tamm plasmon polariton (TPP) has been investigated at the interface between a cholesteric liquid crystal and a metasurface. Different from conventional TPP that occurs with distributed Bragg reflectors and metals, the chiral–achiral TPP is successfully demonstrated. The design of the metasurface as a reflective half-wave plate provides phase and polarization matching. Accordingly, a strong localized electric field and sharp resonance are observed and proven to be widely tunable.

## 1. Introduction

**Figure 1:**
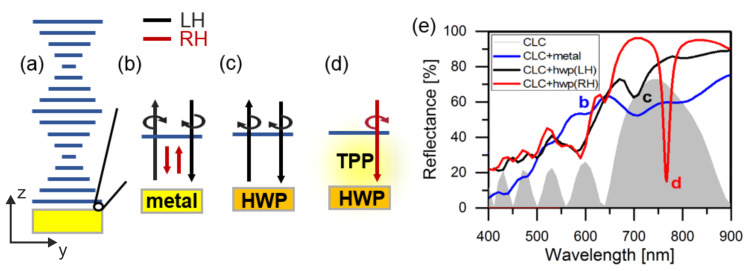
(**a**) Schematic of the right-handed helical CLC combined with a bottom film. Polarization dynamics of the light between (**b**) CLC and a metal mirror and (**c**,**d**) CLC and a half-wave plate metasurfaces. (**e**) Reflectance spectra of CLC–metal and CLC-half-wave plate simulated by the software for Multiphysics simulation COMSOL. The rotation arrow direction indicates the right-handed (RH) and the left-handed (LH) circular polarizations propagating along the z axis. The center of the CLC stopband is at 750 nm. The refractive index of the SiO_2_ layer is fixed at 1.45, and that of the gold nanobrick and PMMA layer comes from the databases of Johnson and Christy [21] and Sultanova [22], respectively. The full reflectance spectra of the measurements is presented in the Supplementary Materials.

In recent years, metamaterials have been widely utilized in photoelectronics due to the advances in controlling the phase, polarizations, and chirality. Indeed, chirality gives an additional degree of freedom in photonic systems. Therefore, chiral photonics has received a lot of attention lately, such as chiral-selective metamirrors [1,2,3,4], chiral quantum optics [5], spectropolarimetry [6], etc. Chiral properties can be effectively enhanced using metamaterials and photonic-crystal cavities. In the literature, chirality surface states could be observed on the surface of topological materials [7] or at the interface of two cholesteric liquid crystals (CLCs) [8,9]. However, it would be difficult to be observed at a chiral–achiral interface as the polarization state could not be preserved. An example of such an interface state is Tamm plasmon polariton (TPP). It was first proposed in 2007 [10] and is similar to the Tamm state in a semiconductor, where electrons are localized at the surface of the crystal. The TPP appears between the metal and the periodic dielectric of high and low refractive indices, which is called a distributed Bragg reflector (DBR). Later it was shown that this state can be utilized for absorbers [11], sensors [12,13,14], Tamm plasmon lasers [15,16,17], and solar cells [18,19]. The excitation of chiral-selective TPP at the interface between a CLC and a flat metal film is impossible (please see Figure 1a,b), and the resonance dip cannot be seen within the CLC stopband [20] unless the polarization of reflected light from the metal is changed. Then, the high reflection of the CLC stopband can be maintained without the localization of the light at the interface between the CLC and the metal.

In this regard, a novel design combining a CLC and a half-wave plate metasurface is proposed. The possibility of excitation of a chiral TPP in this structure is demonstrated experimentally for the first time and confirmed numerically. The tuning of the chiral-TPP wavelength is shown by varying the temperature.

## 2. Description of the Model

**Figure 2:**
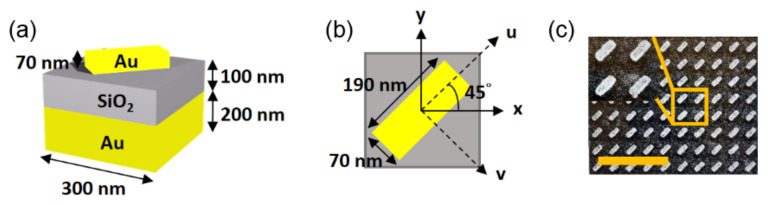
(**a**,**b**) Schematic of a unit cell of metasurface consisting of an Au-SiO_2_-Au structure. The dashed axes of “u” and “v” define the longer and shorter axes of the nanobrick. The “x” axis defines the CLC director at the surface; it is oriented at $\chi =45^{\circ }$. (**c**) The scanning electron microscopic image (the scale bar is 1.2 µm). The detailed images of the nanobricks are shown in the inset.

Figure 1b shows that partial right-handed circularly polarized light is transmitted through the right-handed CLC layer of a finite thickness, and the polarization changes when reflected from the metal. The reflected light with left-handed circular polarization passes through the CLC, and the reflectance spectra correspond to the blue line in Figure 1e, resembling the combination of reflection spectra of only metal and CLC. In order to preserve the reflected circular polarization, a quarter-wave plate was proposed to match the phase [23]. As the partial right-handed circular polarization transforms into linear polarization when light passes through the quarter-wave plate, the polarization of the reflected light remained unchanged. Hence, right-handed circularly polarized light is localized between the CLC and metal, yielding resonance. However, in reality, a typical quarter-wave plate is much thicker than the wavelengths, which could not sustain the surface waves. For example, a 75 µm thick conventional quarter-wave plate (Edmund Optics) has been tested and it was found that the interference diminishes the TPP resonance. Here, we replace the thick phase plate by a metasurface, with the function of a reflective half-wave plate (HWP), as shown in Figure 2. The dimensions of the unit cell with a nanobrick fabricated using electron beam lithography are shown (please see Supplementary Materials). The SiO${}_{2}$ layer and bottom metal film are used to control the phase of the reflected light [24,25,26,27,28,29]. This design allows the handedness of reflected light to be preserved and the polarization-matching condition fulfilled. In this paper, the experimentally measured Q factor of the chiral-selective TPP is 27.2. The Q factor of the TPP resonance is conventionally determined by the losses and the volume of the resonator. In our case, the cavity volume is mainly governed by the CLC thickness. In addition, loss is predominantly due to the absorption in the plasmonic metasurface. Alternatively, the all-dielectric structures allowed us to obtain higher Q values at the expense of compactness. Additionally, the experimental implementation of all-dielectric handedness-preserving structures is difficult [30].

The phase matching is another crucial condition for localized state excitation. This second condition is equivalent to the geometric phase condition for the angle between the long axis of the metasurface nanobrick and the CLC direction [31,32]. In Figure 2b, this angle is shown to be $\chi =45^{\circ }$ and the TPP resonance frequency approximately corresponds to the center of the CLC stopband. By varying, the frequency can be easily tuned through the entire CLC stopband and even switched off [31]. This possibility is provided by the junction of two mirrors with unique and complementary properties. The CLC is chiral and the metasurface is achiral hence anisotropic. The CLC is fluidic and tunable and the metasurface serves as a robust solid basement for tunability. Such chiral tunability is intensively investigated in self-organized structures related to tensagrity, durotaxis and phototropism [33,34].

## 3. Results

**Figure 3:**
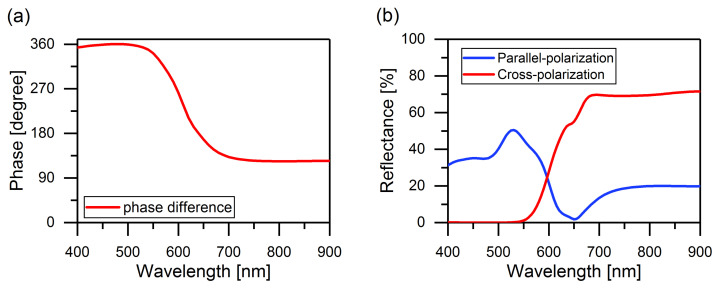
(**a**) Phase difference for reflection from the half-wave plate, with the phase for u-polarized light subtracts the phase of v-polarized light and (**b**) amplitude of circular cross-polarized reflected light, conventional mirror behavior (blue) and co-polarized reflected light, HWP behavior (red) simulated by the software for Multiphysics simulation COMSOL.

The phase difference in reflection is defined as the phase of the u-polarized light subtracting the phase of the v-polarized light (Figure 3a). It is assumed that the light is incident on the metasurface from the air. The phase difference approaches π in the wavelength range from 700 to 900 nm, which satisfies the properties of a half-wave plate [35,36,37]. Due to the phase change, the direction of the rotation is opposite when circularly polarized light impinges on the half-wave plate. Therefore, the handedness remains unchanged between the reflected light and the incident light.

For the incidence of right-handed circular light, the electric field of the reflection in circular basis can be written as [38]:

(1) $$E_{\mathrm{ref}}=\frac{1}{2}\left[\left(r_{u}-r_{v}\right)e^{-2i\chi };\left(r_{u}+r_{v}\right)\right].$$

Here, χ is the angle between the x and u axis, and $r_{u}$ and $r_{v}$ are the complex reflection coefficients for the u and v axes, respectively. To obtain the unchanged polarization of the reflected light, only the first term of Equation (1) is considered. Therefore, the dimensions of the nanobrick were adjusted to minimize $r_{u}+r_{v}$ and maximize $r_{u}-r_{v}$. Figure 3b shows the amplitude of the unchanged (co-polarized) and opposite (cross-polarized) handedness of the reflected light. In this case, at a wavelength of 650 nm, a perfect reflection was observed, since the reflection coefficient for co-polarized light was close to 0. Moreover, the HWP effect sharply decreased to the left from the resonance; therefore, the CLC stopband center $\lambda_{0}=750$ nm was chosen to the right from HWP resonance.

According to the temporal coupled-mode theory [39] applied to chiral Tamm state [9], the resonance is described by the reflection amplitude of the total CLC–HWP structure:

(2) $$r_{TP}\left(\omega \right)=1-\frac{2\gamma_{1}}{i\left(\omega_{0}-\omega \right)+\left(\gamma_{1}+\gamma_{2}\right)},$$

where $\gamma_{1}=2kc\exp\left(-2knL\right)$, $\gamma_{2}=\left(1-R\right)kc/2$, $k=\pi \delta \sin2\chi /\lambda_{0}$, $\lambda_{0}/p=n+\sqrt{n^{2}-1}$ cos2χ, $\omega_{0}=2\pi c/\lambda_{0}$ is resonant cyclic frequency, χ is the angle between the u axis of the HWP–metasurface and the cholesteric director at the interface with the HWP-metasurface, $0^{\circ }<\chi <90^{\circ }$, *L* is the cholesteric layer thickness, *R* is the co-handed reflectance of metasurface, for the cholesteric *p* is the pitch, $n=\sqrt{n_{e}^{2}-n_{o}^{2}}/2$ is the average refractive index and $\delta =\left(n_{e}^{2}-n_{o}^{2}\right)/\left(n_{e}^{2}+n_{o}^{2}\right)$ is anisotropy.

A novel design combining a CLC and a half-wave plate metasurface is proposed (see Figure 1c,d). Between the CLC and the metasurface, poly(methyl methacrylate) (PMMA) is coated as a layer of 480 nm in thickness to protect the metasurface. Alignment of the CLC is in the direction of the x axis on both the top substrate and bottom protecting layer by a surface rubbing machine. The bonding process combines the superstrate and PMMA–metasurface. Then, CLC was injected into the gap by capillary action, and the thickness of the gap was 1.5 m. The CLC used has ordinary and extraordinary refractive indices of $n_{o}$ = 1.52 and $n_{e}$ = 1.75, respectively. The center wavelength $\lambda_{c}$ of the CLC stopband was 750 nm, as calculated by the equation $\lambda_{0}=p\langle n\rangle$, where *p* is the helical pitch and 〈n〉 is the average refractive index of the CLC. The optical axis of the CLC lies on the *x*-*y* plane, where the orientation depends on the position of the z axis along the helical pitch of the liquid crystal. The equivalent permittivity of the dielectric tensor from CLC can be written as [40]:

(3) $$\varepsilon =\varepsilon_{0}\left[\begin{array}{ccc} \bar{\varepsilon }+\frac{1}{2}\Delta \varepsilon \cos\left(\frac{4\pi z}{p}\right) & \frac{1}{2}\Delta \varepsilon \sin\left(\frac{4\pi z}{p}\right) & 0\\ \frac{1}{2}\Delta \varepsilon \cos\left(\frac{4\pi z}{p}\right) & \bar{\varepsilon }+\frac{1}{2}\Delta \varepsilon \cos\left(\frac{4\pi z}{p}\right) & 0\\ 0 & 0 & \bar{\varepsilon }+\frac{1}{2}\Delta \varepsilon \end{array}\right].$$

Here, $\bar{\varepsilon }=\left(n_{e}^{2}+n_{o}^{2}\right)/2$, $\Delta \varepsilon =(n_{e}^{2}-n_{o}^{2})$, and z represents the position along the helical axis of the planar CLC. Modeling was conducted by using the finite-element method software COMSOL Multiphysics 4.3b and verified by the Berreman matrix method [41].

**Figure 4:**
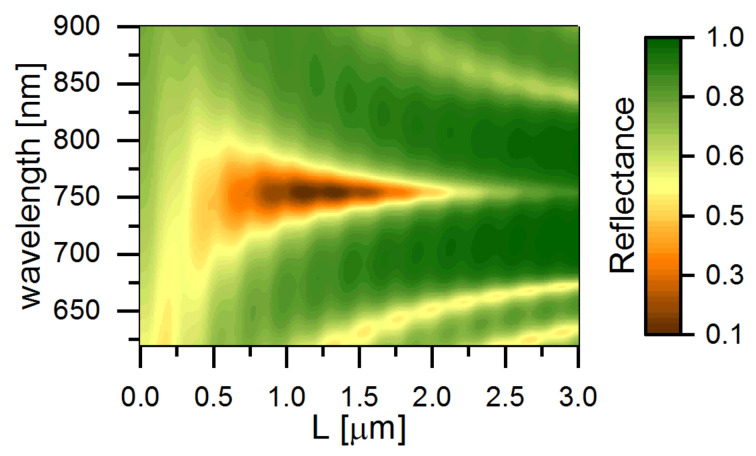
Reflectance spectra of the structure versus the cholesteric layer thickness *L* simulated by the Berreman method. The incident light is right-handed circular polarized (Figure 1b). Dark dip in the center is the TPP resonance. Reflectance minimum corresponds to the optimal coupling with equal losses through the cholesteric and the metasurface. The resonance bandwidth decreases with increasing *L*. Small resonances are observed at the edges of cholesteric stopband. Vertical periodic ripples are caused by additional reflection from the upper cholesteric interface; the rippling period equals the half-pitch of cholesteric helix.

The reflectance spectrum of Figure 4 was obtained through the Berreman method simulation. It is in good agreement with Equation (2) for *R* = 0.7. When $\gamma_{1}\left(L\right)=\gamma_{2}\left(R\right)$, exp(−2nkL)=2(1−R), the optimal cholesteric thickness is *L* = 1.5 µm.

As for the design shown in Figure 1c, the most important point is that the polarization of the reflected light remains the same as that of the incident light, which provides the phase and polarization matching. As illustrated in Figure 1c,d, the energy of light would be localized between the CLC and the half-wave plate to achieve the resonance condition. Therefore, in Figure 4, a narrow reflection dip is clearly observed within the optical stopband of the CLC.

**Figure 5:**
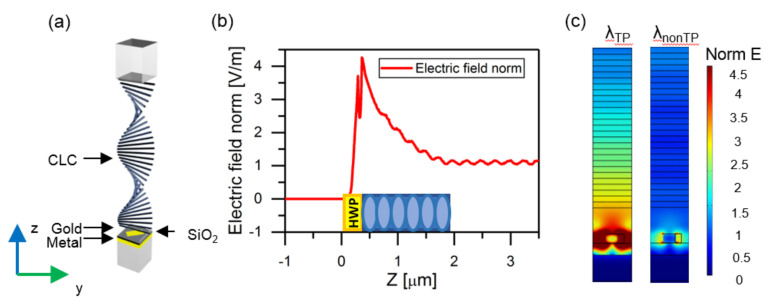
(**a**) Structure of the CLC combined with metasurfaces. (**b**) The maximum of the electric field near CLC–metasurface interface. (**c**) Distribution of normalized electric fields at the resonant and nonresonant wavelengths. Field distribution at the resonant wavelength in *x*-*y* plane is shown in the Supplementary Materials.

As shown in Figure 5, near-field analysis indicates that the strong electric field is localized at the interface [42] between the CLC and the metasurface at the resonance wavelength. In contrast, when the wavelength is nonresonant, the electric-field distribution acts as at an ordinarily-reflecting mirror. The maximum amplitude of the localized electric field at the resonant wavelength is approximately four times larger than that at a nonresonant wavelength. At any point inside the structure the mode has two running wave components with almost equal amplitudes. In Figure 5b, the electric field profile shows spatial ripples due to the interference between the running waves. The ripple period is half-wave. In contrast to a conventional Tamm mode, the interference makes no nodes or antinodes as both running waves are right-handed circularly polarized. In other words, every x-polarized node coincides with a y-polarized antinode and vice versa, which results in a smooth profile [30]. The nontrivial field profile in CLC was thoroughly investigated and illustrated in [43].

The other advantage of using CLC to generate chiral-selective TPP is that CLC could be easily controlled by external stimuli such as electric field [44] and ambient temperature [45]. By utilizing the temperature dependence of the CLC stopband, the wavelength of resonance can be effectively controlled to achieve a wide-range tunability [46].

**Figure 6:**
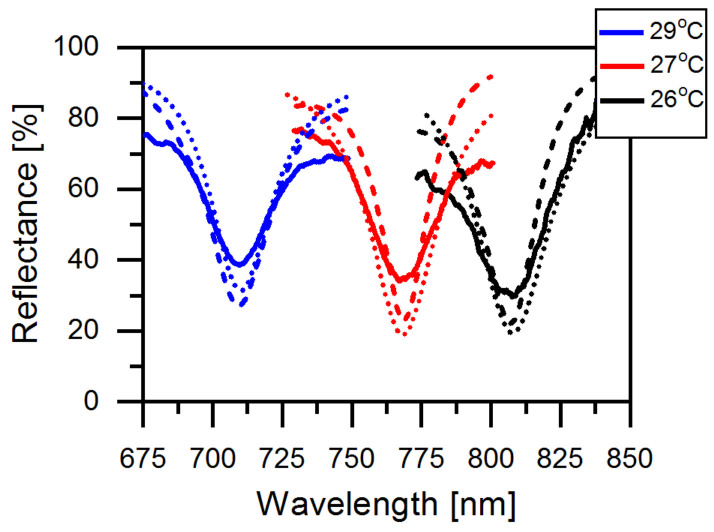
Reflectance spectra of the CLC–metasurface simulated by Berreman method (dotted lines); COMSOL (dashed lines) and experiment results (solid lines) by increasing the temperature from 26 ${}^{\circ }$C to 29 ${}^{\circ }$C.

As shown in Figure 6, with an increase in temperature from 26 to 29 ${}^{\circ }$C, the resonance dip shifts to a shorter wavelength due to the movement of the CLC stopband. The simulation (dashed lines) and experimental (solid lines) results are in reasonably good agreement. The deviation may result from the thickness of the CLC layer to be different from the setting in the simulated model due to its changes within the measurement area. The imperfection of the rubbing on the PMMA may be an additional reason for the discrepancy between the measured and simulated data. The simulated temperature dependence of the helix pitch was considerably tuned to satisfy Table 1. This restricted our prediction ability for the first experiment. The widening in resonance bandwidth might be due to the extra loss in metal during the nanofabrication process. Table 1 manifests the tunability of the resonance wavelengths of TPP and CLC stopbands with varying temperatures. The difference between the resonant wavelength $\lambda_{TP}$ and the center wavelength $\lambda_{0}$ is presumably due to the thin protecting layer. The quality factor obtained by coupled mode theory is $Q=\omega_{0}/2\left(\gamma_{1}+\gamma_{2}\right)\approx$ 27.2, which is in good agreement with the experimental data.

## 4. Conclusions

In conclusion, we demonstrated that chiral-selective TPP can be successfully excited at the interface between metasurface of reflective half-wave plate and CLC. This photonic surface state combines properties of both anisotropic metasurface and chiral CLC, providing a wide-ranging orientational tunability. A strong localized electric field at the interface between the CLC and the metasurface was observed. Furthermore, by changing the center wavelength of the stopband of the CLC with different pitches and temperatures, the resonance wavelength of TPP was tuned flexibly. This device can potentially be applied to optical switches and polariton lasers.

## Figures and Tables

**Table 1:** Tunability of the resonant wavelength of chiral-selective TPP ( $\lambda_{TP}$), center wavelength of CLC stopband ( $\lambda_{0}$), pitch *p* and *Q* factor of the TPP with respect to the different temperatures.

	26 ${}^{\circ }$C	27 ${}^{\circ }$C	29 ${}^{\circ }$C
$\lambda_{TP}$ (nm)	809	767	709
$\lambda_{0}$ (nm)	890	805	665
*p* (nm)	495.4	468.5	428.1
*Q* factor	28.5	27.2	26.7

## Data Availability

The data presented in this study are available upon reasonable request from the corresponding author.

## Supplementary Materials

The following are available online at https://www.mdpi.com/article/10.3390/ma14112788/s1, Figure S1: Fabrication process of metasurfaces, Figure S2: (a,b) Structure of the bonding devices of CLC and metasurface, which are composed of two plywoods and four precision screws, Figure S3: Schematic of sample fabrication procedure. The plano-convex lens is used as the superstrate to control the thickness of cell gap, and CLC is poured into the gap between the lens and metasurface by capillary action, Figure S4: Experimental setup for reflection measurement. LS: Halogen light source; PH: pinhole; P: polarizer (Thorlabs, WP25M-UB); WP: quarter-wave plate (Edmund, 75 µm in thickness); BS: cube beam-splitter (Thorlabs, BS016); Obj: objective (Olympus, LMPLFLN 50x); S: sample of CLC-metasurface; Detector: spectrometer (Ocean, USB2000+), Figure S5: (a) Measured reflectance for the co-(black line) and cross-polarized (red line) light at normal incidence. Optical image of metasurfaces with (b) cross-polarized measurement and (c) co-polarized measurement, Figure S6: The experimental and simulated reflectance spectra of the structure, Figure S7: Ex (left) and Ey (right) components of the electric field in x-y plane.
