# Supplementary material for: Chiral-Selective Tamm Plasmon Polaritons
Source: Materials (Basel). 2021 May 24;14(11):2788. doi: 10.3390/ma14112788 (PMC8197218; doi:10.3390/ma14112788)
Supplement: Supplementary file 1 [file materials-14-02788-s001.zip › materials-1213960-supplementary.pdf]

materials

MDPI

*Supplementary Information*

# Chiral-Selective Tamm Plasmon Polaritons

**Meng-Ying Lin [1,2], Wen-Hui Xu [1,2], Rashid G. Bikbaev [3,4,*], Jhen-Hong Yang [5,6], Chang-Ruei Li [7,8], Ivan V. Timofeev [3,4], Wei Lee [1,2] and Kuo-Ping Chen [1,2,*]**

1 Institute of Imaging and Biomedical Photonics, College of Photonics, National Chiao Tung University, 301 Gaofa 3rd Road, Tainan 71150, Taiwan; e1253400@gmail.com (M.-Y.L.); uxxsh8@gmail.com (W.-H.X.); wlee@nctu.edu.tw (W.L.)
2 Institute of Imaging and Biomedical Photonics, College of Photonics, National Yang Ming Chiao Tung University, 301 Gaofa 3rd Road, Tainan 71150, Taiwan
3 Kirensky Institute of Physics, Federal Research Center KSC SB RAS, 660036 Krasnoyarsk, Russia; ivan-v-timofeev@ya.ru
4 Siberian Federal University, 660041 Krasnoyarsk, Russia
5 Institute of Photonic System, College of Photonics, National Chiao Tung University, 301 Gaofa 3rd Road, Tainan 71150, Taiwan; s87069@hotmail.com
6 Institute of Photonic System, College of Photonics, National Yang Ming Chiao Tung University, 301 Gaofa 3rd Road, Tainan 71150, Taiwan
7 Institute of Lighting and Energy Photonics, College of Photonics, National Chiao Tung University, 301 Gaofa 3rd Road, Tainan 71150, Taiwan; g10592000@hotmail.com
8 Institute of Lighting and Energy Photonics, College of Photonics, National Yang Ming Chiao Tung University, 301 Gaofa 3rd Road, Tainan 71150, Taiwan
* Correspondence: bikbaev@iph.krasn.ru (R.G.B.); kpchen@nctu.edu.tw (K.-P.C.)

**Citation:** Lin, M.-Y.; Xu, W.-H.; Bikbaev, R.G.; Yang, J.-H.; Li, C.-R.; Timofeev, I.V.; Lee, W.; Chen, K.-P. Chiral-Selective Tamm Plasmon Polaritons. *Materials* **2021**, *14*, 2788. https://doi.org/10.3390/ma14112788

Academic Editor: Maria Principe

Received: 23 April 2021
Accepted: 21 May 2021
Published: 24 May 2021

**Publisher's Note:** MDPI stays neutral with regard to jurisdictional claims in published maps and institutional affiliations.

**Copyright:** © 2021 by the authors. Submitted for possible open access publication under the terms and conditions of the Creative Commons Attribution (CC BY) license (http://creativecommons.org/licenses/by/4.0/).

## 1. Sample Fabrication

### *1.1. E-beam Lithography for Metasurface*

E-beam lithography is used to fabricate the metasurface. First, the 200 nm gold film and 100 nm amorphous $SiO_2$ are deposited by sputter on the glass substrate (see Figure S1). Then, the PMMA A4 photoresist with thickness equals to 200 nm is spin coated on the substrate at 1500 rpm for 15 s and 8000 rpm for 30 s, respectively. Then it is prebaked at 180°c for 90s. The lower spin speed is for flatness, and higher spin speed is for controlling thickness. E-beam lithography system (ELS-7500EX, ELIONIX) is used to define the pattern, and the photoresist is developed by developers (MIBK:IPA = 1:3) for 30 s. Then, 40 nm gold film is deposited by electron-gun evaporation system (VT1-10CE, ULVAC) with 0.5 Å/s deposition rate in $5 \times 10^{-6}$ Torr. The sample is immersed in acetone liquid for lift-off residual photoresist and unnecessary gold film.

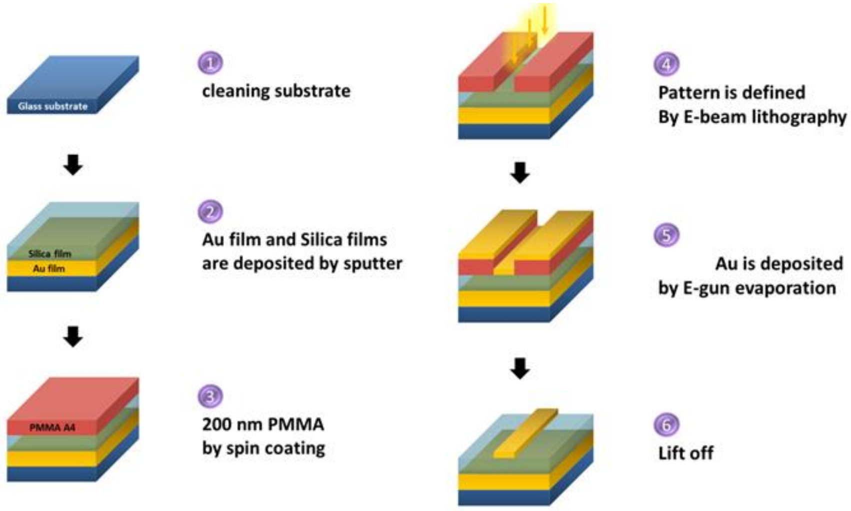


**Figure S1.** Fabrication process of metasurfaces.

*1.2. Fabrication of CLC-metasurface Devices with Small Cell Gaps*

The assembly of CLC and the metasurface is introduced in Figure S2. The main purpose of the design is to overcome the limit of the thickness for the conventional LC cell gaps. It can give more tunable range of thickness due to the requirement that the cell gaps of CLC cannot be too thick; otherwise the optical Tamm state cannot be excited. Therefore, the plano-convex lens with the focal length as 5000 mm is used as the top substrate (superstrate) in order to provide small gaps between superstrates and substrates. The bonding devices combine two plywoods and 4 precision screws to bond the sample of CLC and metasurface, and the side view from bottom to top is plywood-substrate- metasurface-spacer layer-CLC-plano convex lens-plywood.

Schematic of the sample fabrication is shown in Figure S3, and PMMA with 480 nm thickness is coated as a protective layer on the metasurfaces at 1000 rpm for 15 s and 1500 rpm for 30 s, respectively. Surface alignment of PMMA is rubbed in x-direction by rubbing machine. The bonding process combines plano-convex lens and PMMA-metasurface, where the focal length of lens is 5000 mm. Then, CLC is poured into the gap by capillary action, and the thickness of the gap is 3 pitches for CLC (~1.5 μm in cell gaps), and the thickness of the gap is verified by the fitting of the interference dip. The liquid crystal cell was placed into the thermostatic cuvette to measure the reflection spectra in the temperature range of 20–40 °C with a stabilization accuracy of ±0.2 °C.

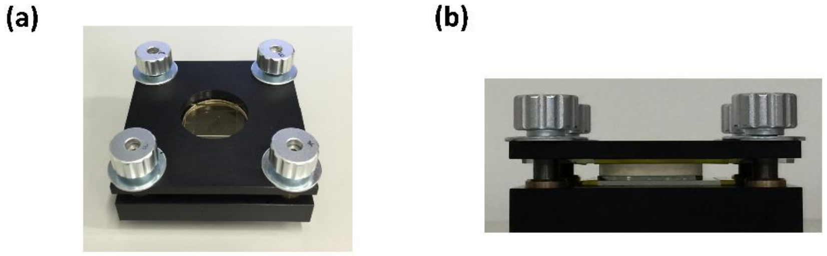


**Figure S2.** (**a**,**b**) Structure of the bonding devices of CLC and metasurface, which are composed of two plywoods and four precision screws.

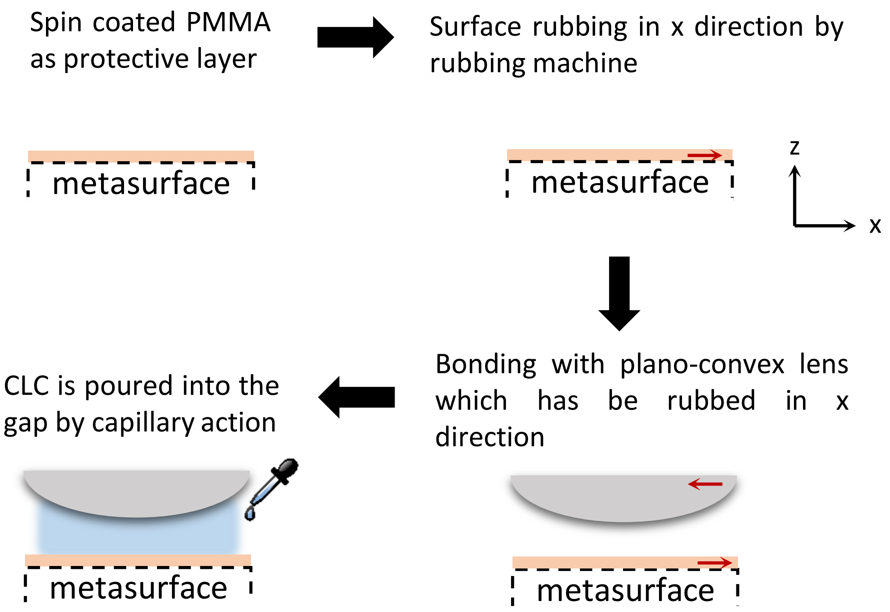

**Figure S3.** Schematic of sample fabrication procedure. The plano-convex lens is used as the superstrate to control the thickness of cell gap, and CLC is poured into the gap between the lens and metasurface by capillary action.

## 2. Measurement Setup

Experimental setup for spectra measurement has been shown in Figure S4 by using reflection microscopy (Olympus BX51). The broadband light source of the halogen lamp initially passes through a pinhole to reduce the beam size due to the dimension of die on sample is only 100 × 100 μm. Then, the linear polarizer and quarter-wave plate is used to transform the light into circular polarization by rotating 45 degrees between polarization of polarizer and fast axis of quarter-wave plate. Therefore, no matter what kinds of polarization, it can be achieved by these two components. Then, light reflected from beam splitter focuses on sample with 50× objective, and reflected light is received by spectrometer finally. The reason why using pinhole and 50× objective instead of 100× objective is due to the depth of field (DOF), that is inadequate for the thickness of the sample.

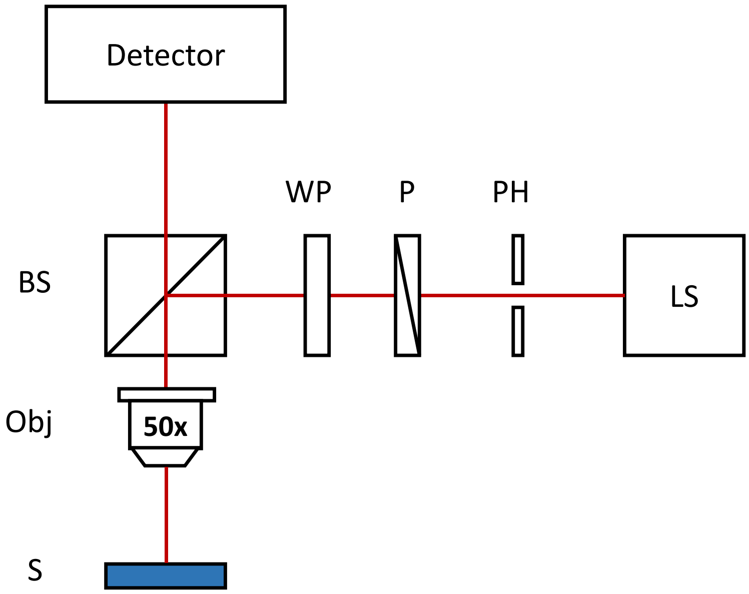


**Figure S4.** Experimental setup for reflection measurement. LS: Halogen light source; PH: pinhole; P: polarizer (Thorlabs, WP25M-UB); WP: quarter-wave plate (Edmund, 75 μm in thickness); BS: cube beam-splitter (Thorlabs, BS016); Obj: objective (Olympus, LMPLFLN 50×); S: sample of CLC-metasurface; Detector: spectrometer (Ocean, USB2000+).

## 3. Characterization of Metasurfaces

As shown in Figure S5, the cross-polarized reflection approaches 60% and co-polarized reflection is less than 10% at the wavelength of near-infrared. The phenomenon of transformation is evidence that the optical image is shown in Figure S5b,c. The die is bright in Figure S5b due to the high reflection of cross-polarized light comparing to the image of co-polarized light in Figure S5c. Therefore, the circular polarized reflection light from metasurface could remain the same comparing to the incident light.

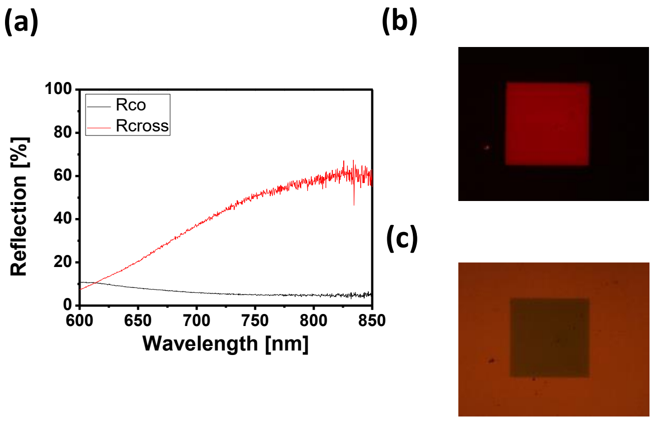


**Figure S5.** (**a**) Measured reflectance for the co-(black line) and cross-polarized (red line) light at normal incidence. Optical image of metasurfaces with (**b**) cross-polarized measurement and (**c**) co-polarized measurement.

## 4. Simulation Result

The experimental and simulated reflectance spectra of the structure in the range from 400 to 850 nm are shown in Figure S6.

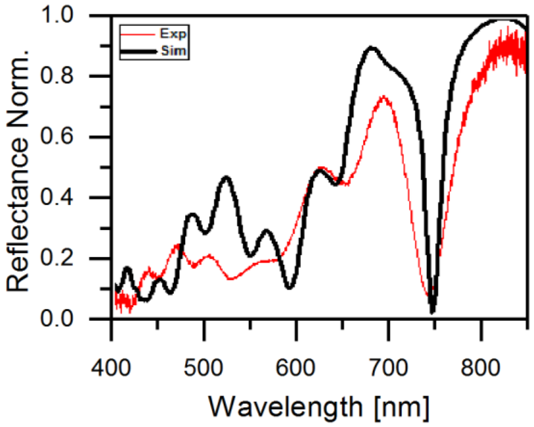


**Figure S6.** The experimental and simulated reflectance spectra of the structure.

Field distribution at the chiral-TPP wavelength in *x*-*y* plane is shown in figure S7.

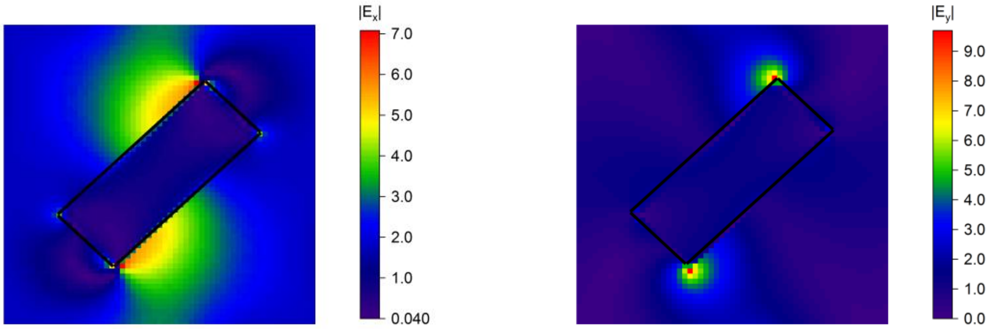


**Figure S7.** $E_x$ (**left**) and $E_y$ (**right**) components of the electric field in *x*-*y* plane.
